# The Roles of Tissue-Resident Memory T Cells in Lung Diseases

**DOI:** 10.3389/fimmu.2021.710375

**Published:** 2021-10-11

**Authors:** Rui Yuan, Jiang Yu, Ziqiao Jiao, Jinfei Li, Fang Wu, Rongkai Yan, Xiaojie Huang, Chen Chen

**Affiliations:** ^1^Xiangya School of Medicine, Central South University, Changsha, China; ^2^Department of Oncology, The Second Xiangya Hospital of Central South University, Changsha, China; ^3^Department of Radiology, Johns Hopkins University School of Medicine, Baltimore, MD, United States; ^4^Department Cardiovascular Surgery, The Second Xiangya Hospital of Central South University, Changsha, China; ^5^Department of Thoracic Surgery, The Second Xiangya Hospital of Central South University, Changsha, China; ^6^Hunan Key Laboratory of Early Diagnosis and Precise Treatment of Lung Cancer, The Second Xiangya Hospital of Central South University, Changsha, China

**Keywords:** tissue-resident memory T cells, non-small-cell lung cancer, lung infection, immunotherapy, vaccine

## Abstract

The unique environment of the lungs is protected by complex immune interactions. Human lung tissue-resident memory T cells (T_RM_) have been shown to position at the pathogen entry points and play an essential role in fighting against viral and bacterial pathogens at the frontline through direct mechanisms and also by orchestrating the adaptive immune system through crosstalk. Recent evidence suggests that T_RM_ cells also play a vital part in slowing down carcinogenesis and preventing the spread of solid tumors. Less beneficially, lung T_RM_ cells can promote pathologic inflammation, causing chronic airway inflammatory changes such as asthma and fibrosis. T_RM_ cells from infiltrating recipient T cells may also mediate allograft immunopathology, hence lung damage in patients after lung transplantations. Several therapeutic strategies targeting T_RM_ cells have been developed. This review will summarize recent advances in understanding the establishment and maintenance of T_RM_ cells in the lung, describe their roles in different lung diseases, and discuss how the T_RM_ cells may guide future immunotherapies targeting infectious diseases, cancers and pathologic immune responses.

## Introduction

Tissue-resident memory T (T_RM_) cells comprise a recently identified lymphocyte lineage that occupies tissues without recirculating. They reside in epithelial barrier tissues, such as lung, gastrointestinal tract, reproductive tract, and skin, and in some non-barrier tissues, such as brain, kidney, and joint (1–3). T_RM_ cells are transcriptionally, functionally and phenotypically distinct from circulating effector memory T cells (4).

The human lung is continuously exposed to environmental and microbial antigens (2). T_RM_ cells in lung tissues play a crucial role in both innate and adaptive immune responses to lung infections, such as Respiratory Syncytial Virus (RSV), SARS-CoV-2, Brucella and Mycobacterium tuberculosis (5–7). Growing evidence has revealed that the T_RM_ cells reside in tissues in the absence of antigens and may provide rapid on-site immune protection against previously exposed pathogens in peripheral tissues to accelerate pathogen clearance (5).

Recently, T_RM_ cells have been found to participate in anti-tumor immunity as well. T_RM_ cells can promote intra-tumoral cytotoxic T lymphocytes (CTLs) responses and are correlated with overall survival in lung cancer patients (8–10). Induction of T_RM_ cells can enhance the efficacy of cancer vaccines (11) and increase the response rate when using anti-PD-1 antibodies to reverse tumor-induced T cell exhaustion in NSCLC patients (12, 13).

In addition to the protective roles against diseases, evidence suggests that T_RM_ cells also become activated after sensitization to self-antigens. Aberrantly activated T_RM_ cells can induce autoimmune disorders, such as autoimmune hepatitis and psoriasis (4, 14). In the respiratory system, T_RM_ specifically activated by environmental allergens might underlie the development and worsening of allergic asthma and other airway diseases (15–18). Pathogenic T_RM_ cells may contribute to chronic pulmonary inflammation and fibrosis (2). T_RM_ cells are also recognized as the primary mediator of acute cellular rejection (ACR) after lung transplantation.

This review aims to comprehensively summarize the current understandings of the biology of T_RM_ cells, including the distinguishing molecular markers, regulators, and functions of T_RM_ cells, discuss the contributions of T_RM_ cells to lung diseases, especially infectious diseases and tumors, and highlight potential T_RM_-related therapeutic strategies for respiratory diseases.

## Defining Lung T_RM_ Cells

Human memory T cells can be broadly categorized into three subsets: central memory T cells (T_CM_), effector memory T cells (T_EM_), and tissue-resident memory T cells (T_RM_). T_CM_ cells are memory T cells that recirculate through secondary lymphoid organs, whereas T_EM_ cells recirculate through nonlymphoid tissues. T_RM_ cells, by contrast, typically reside in specific tissues, especially mucus organs, such as lungs and gastrointestinal tracts, defending against pathogens in peripheral nonlymphoid tissues. Compared with T_CM_ and T_EM_ cells, commitment to the tissue of residence is a defining characteristic of T_RM_ cells, which has been described in almost all organs (19). Most memory T cells in non-lymphoid tissues are T_RM_ cells, either CD4+ or CD8+ (9). T_RM_ cells can be further fractionated by their functional characteristics into epithelial and stromal T_RM_ cells (20, 21).

T_RM_ cells are widely distributed throughout the body, including the skin, lungs, lymphoid organs, etc. However, T_RM_ cells in different organs display distinct properties (21). In healthy human skin, most of the T_RM_ cells are dermal CD4+CD69+CD103− cells, which express high levels of the cutaneous lymphocyte antigen (CLA) and specific chemokine receptors like CCR4 (22). With increased expression of T cell factor-1 (TCF-1) and lymphoid enhancer factor-1 (LEF-1), human lymph node CD8+ T_RM_ cells exhibit a phenotype of tissue residency as well as an organ-specific signature (23). Human lymph node-specific profile of memory CD8+ T cells is defined by expression of CXCR5 and TCF-1 and high proliferative capacity, which accordingly indicates that human lymph node memory CD8+ T cells display higher proliferative capacity than their counterparts in other tissues (24). Similarly, T_RM_ cells in non-small-cell lung cancer (NSCLC) may be identified by CD39 and CD103 (25). In mice, the formation and maintenance of skin T_RM_ cells is mediated by chemokine receptors like CXCR3, CXCR6, and CCR10 (26). Genetic knockout studies of mice have shown that CD69 deficiency reduces the retention of CD4+ T cells in the bone marrow (27).

Eomes and T-bet are T-box transcription factors (TFs) that restrict the formation of CD103+T_RM_ cells, indicating that downregulation of both transcription factors is crucial for the generation of CD103+ T_RM_ cells. Eomes TF is significantly downregulated in CD8+CD103+T_RM_ cells compared to circulating T_EM_ or T_CM_ cells. The residual T-bet expression upregulates interleukin-15 receptor (IL-15R) β-chain (CD122) expression, which is essential for long-term T_RM_ cell survival. The coordinated downregulation of both T-box TFs optimizes cytokine transforming growth factor-beta (TGF-β) signaling, leading to the efficient development of CD8+CD103+T_RM_ cells (28).

Both CD69 and CD103 are expressed in CD8+ T_RM_ cells and less frequently, CD103 is expressed in CD4+T_RM_ cells (29). CD69, which is an early activation marker involved in lymphocyte proliferation and retention, plays a key role in distinguishing T_RM_ cells from those in circulation. Additionally, CD103 is a key to recognize most CD4+and CD8+T_RM_ cells (30). The expression of CD103 helps T_RM_ cells dock to the E-cadherin-expressed on epithelial cells and prevents them from re-circulating in the blood (31). It is generally accepted that TGF-β is an upstream regulator of T_RM_ transformation. Increasing TGF-β *in vivo* appears to significantly increase the number of local T_RM_ cells (32, 33). It has been revealed that the function and expression of CD103 greatly depends on the TGF-β, indicating that the T_EM_ and other T cells might lack TGF-β cytokines and thus fail to upregulate CD103 (34). CD39 is also highly expressed in T_RM_ cells and is associated with higher T_RM_ cells activities and quantity. CD39 could protect cells from apoptosis induced by adenosine triphosphate (ATP). As a transmembrane glycoprotein and extracellular nucleosidase, CD39 is also present in many biological processes such as adenosine regulation, proliferation, and resident transduction signals (28). While most human CD4+T cells express CD69, a portion of them express CD103+at the same time, especially in the lung. The majority of lung CD4+ T_EM_ phenotype cells express the canonical T_RM_ marker CD69. Comparing the gene expression patterns of CD103+T_RM_ cells in lung and T_EM_ cells in the blood, human lung CD4+CD103+T_RM_ cells express higher levels of *ITGAE* (which encodes CD103), *CTLA4*, *KLRC1* (which encodes the inhibitory receptor NKG2A) (35, 36) and ICOS (31). CD4+CD103+T_RM_ cells express deployment-ready mRNAs encoding effector molecules that rapidly respond to pathogens. Human lung T_RM_ cells express lower levels of *S1PR1* (which encodes the S1P receptor) (37), lymph node-homing molecules, *SELL* (which encodes the lymph node-homing receptor CD62L), *KLRG1* (which encodes the activation marker KLRG1) (35), *KLF2* (which drives expression of S1PR1) and *CCR7* (31). Additionally, some genes have different expressions between CD103+T_RM_ cells and peripheral T_EM_ cells, including genes that encode heat-shock proteins (HSPA1A, HSPA7, HSPA2, and HSPD1), transcription factors (EGR2, FOSB, ATF3, RBPJ, EPAS1, and BATF) (31, 35), anti-apoptotic factors (PHLDA1 and BIRC3), the tumor necrosis factor (TNF) receptor signaling family (TRAF1 and TANK) (31), chemokine (XCL1), solute carrier family members mediating amino acid transport (SLC7A5 and SLC1A5), chemokine receptor (CXCR6), transforming growth factor (TGF-β1), interleukin (IL-21R), the ligand for the death receptor Fas (FASLG), adhesion G-protein-coupled receptor (CD97), interferon-γ receptors (IFNGR) (35), and fatty-acid-binding protein (FABP5) (38). All the genes mentioned above have higher expression levels in lung CD103+T_RM_ cells. Characteristically, lung CD4+CD103+T_RM_ cells exhibit high levels of various integrins and adhesion molecules (31, 39). Compared with blood-derived T_EM_ cells, differences in the molecular expression of lung CD103+T_RM_ cells are summarized in **Table 1**.

**Table 1:** The differences in integrins and molecule expression between lung T_RM_ cells and T_EM_ cells.

Classification	Molecules	Function	Expression level in T_RM_ cells compared with T_EM_ cells	Species	References
Intercellular Adhesion Molecule	ICAM2(CD102)	lymphocyte activation	Higher	Human	(31, 35)
	ICAM1(CD54)	lymphocyte activation	Lower	Human	(35)
Chemokine Receptor	CCR5	lymphocyte recruitment	Higher	Human	(35)
	CCR5	lymphocyte recruitment	Higher	Human	(35)
	CCR7	impairing T-cell homing to lymph nodes	Lower	Human	(31)
	CXCR6	T-cell recruitment	Higher	Human	(31, 35, 36)
	CXCR3	T-cell recruitment	Higher	Human	(35)
	CX3CR1	transmigration through endothelial layers	Lower	Human	(35)
Cytotoxic T-Lymphocyte-Associated Protein	CTLA4	inhibitory molecules	Higher	Human	(31, 35)
Immunoglobulin	LAG3	inhibitory molecules	Higher	Human	(35)
Adenosine Receptor	A2AR	inhibitory molecules	Higher	Human	(35)
Interleukin	IL-17	pro-inflammatory cytokines	Higher	Human	(36)
Interferon	IFN-γ	pro-inflammatory cytokines	Higher	Human and Mouse	(31, 36, 38)
Integrin	CD103	retention, adhesion, and migration to tissues	Higher	Mouse	(36, 37)
	CD49a	retention, adhesion, and migration to tissues	Higher	Human	(36)
Other molecules	CD69	retention, adhesion, and migration to tissues	Higher	Human	(36)
	CD97	G-protein-coupled receptor	Higher	Human	(35)
	CD101	inhibitory molecules	Higher	Human	(36)
	CD279(PD-1)	inhibitory molecules	Higher	Human	(36)
	CD272(BTLA)	inhibitory molecules	Higher	Human	(35)
	SPRY1	inhibitory molecules	Higher	Human	(31, 35)

Several transcription factors play critical roles in the proliferation of T_RM_ cells. The activation of the programmed cell death protein 1(PD-1) signal pathway downregulates the expression of Bhlhe40, which supports mitochondria and chromatin production in T_RM_ cells and is thus essential to the proliferation and maintenance of T_RM_ cells (40). Transcription factors such as BATF, NAB1, and NAB2 are also highly expressed in T_RM_ cells. These factors can regulate T cell metabolisms to maintain their survival and reduce the expression of inhibitory phenotypes (41).

## Lung T_RM_ Cells and Protection Against Respiratory Infection

**Figure 1:**
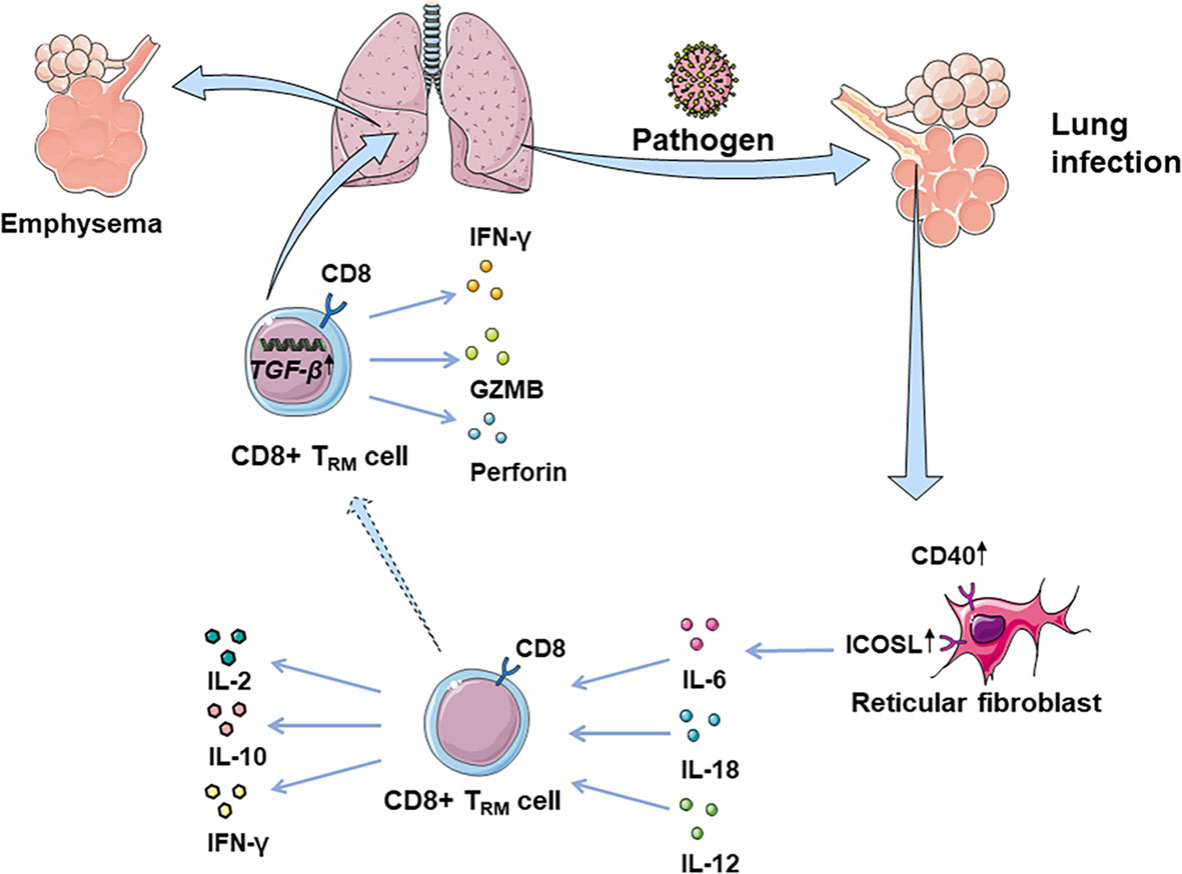
CD8+ T_RM_ cells in lung infection and immunopathology. CD8+ T_RM_ cells are considered as the first line of defense in peripheral tissues against earlier exposure to antigens. CD8+ T_RM_ cells located in the lung parenchyma could rapidly synthesize IFN-γ following the inhalation of pathogens, driven by exposure to IL-12/IL-18. Fibroblast reticular cells located near T cells around the focus can transmit long-lasting activation signals to CD8+T cells, by upregulating ICOSL, CD40, and IL-6. Additionally, CD8+ T_RM_ cells promote the production of IL-2, mobilizing inflammatory response. At the same time, T_RM_ cells can produce IL-10, thus inhibiting the excessive inflammatory response and limiting tissue damage caused by inflammation. However, CD8+ T_RM_ cells can be abnormally deposited in the lung due to overexpression of TGF-β-related genes, which may damage normal tissues by releasing IFN-γ, GZMB, and perforin, leading to lung emphysema or fibrosis.

CD8+T_RM_ cells are considered as the first line of defense in peripheral tissues against pathogens. Many studies suggest that some risk factors may interfere with circulating memory CD8+T cell function (**Table 2** and **Figure 1**). Reticular fibroblasts located near T cells around the infection site can transmit long-lasting activation signals to CD8+T cells by upregulating ICOS ligand (ICOSL), CD40, and interleukin-6 (IL-6), which promotes the preferential differentiation of T cells into T_RM_ cells (55). CD8+T_RM_ cells can produce chemokines after local tissue activation and recruit non-antigen-specific T cells, exerting natural effector functions (56, 57). T_RM_ cells promote the production of IL-2 and some pro-inflammatory cytokines, effectively mobilizing inflammatory responses and exert immune defenses (58). At the same time, T_RM_ cells produce IL-10 and express inhibitory receptors, thus inhibit excessive inflammatory response and limit tissue damage caused by inflammation (59). CD4+ T_RM_ cells in non-lymphoid tissues, such as lung, skin, and genital mucosa, can influence the immune reaction of various pathogenic microorganisms (60–62).

**Table 2:** The features of T_RM_ cells in lung infection or pathological process.

Infection or pathological process	Phenotype	Function or regulation	References
SARS-CoV-2 infection	tissue-resident memory-like Th1 cells and tissue-resident memory-like Th17 cells	Natural Th17 cells were recruited to the infected site by CCL20 on lung epithelial cells stimulated by IL-17A and expanded in the presence of IL-23, which then were converted to T_RM_ cells, existing as ex-Th17 cells and exerting Th1-like immunity in the event of SARS-CoV-2.	(42)
Respiratory Syncytial Virus	CD4+T_RM_ cells and CD8+T_RM_ cells	T_RM_ cells showed gradual differentiation with down-regulated costimulatory molecules and increased CXCR3 expression, which had been implicated in protection against RSV-induced lung pathology in mice *via* dendritic cells and CD8+ T cells.	(43–45)
Bordetella Pertussis	CD69+CD4+T_RM_ cells	T_RM_ cells produced IL-17 and IFN-γ, thereby recruiting neutrophils and preventing their colonization in the nose.	(46–49)
Influenza Viruses	CD8+T_RM_ cells	The expression of PPAR-γ and dendritic cells with high expression of IRF4 can effectively promote the production of CD8+T_RM_ cells which protect the body from influenza viruses by producing IFN-γ and TNF-α.	(50, 51)
Brucella infection	CXCR3lo CD103+CD8+T_RM_ cells and CXCR3hi CD103+CD8+T_RM_ cells	CXCR3hi T_RM_ cells could not be depleted by anti-CD8 mAb, thus inducing protection against Brucella more efficiently.	(52)
Pulmonary Inflammation	CD69hiCD103loCD4+T_RM_ cells	Enhance the secretion of IL-5 and IL-13 which can cause pulmonary inflammation and fibrosis.	(15)
	CD69hiCD103hiCD4+T_RM_ cells	Improve the fibrosis reaction caused by pulmonary inflammation and reduce lung injury.	(15)
Asthma	Th2-T_RM_ cells	Th2-T_RM_ cells expressing high levels of CD44 and ST2 can reside in lung tissues and retain allergen memory. Once re-exposed to an allergen, Th2-T_RM_ cells proliferate near the airway, producing type 2 cytokines that enhance eosinophil activation and promote peribronchial inflammation.	(18, 53, 54)

### Antivirus Effect

In lungs, follicular tissue-resident CD4+ T helper cells contribute to the defense against virus in conjunction with CD8+ T cells relying on IL-21 (63). These helper T cells can also induce antiviral B cell reactions in bronchus lymphoid tissue in flu virus infection, indicating that T_RM_ cells could promote the protective response of B cells and CD8+T cells in lung infections (63). CD8+T_RM_ cells in the lung act as protective agents against viruses through interferon-γ (IFN-γ) (64). Studies of Coronavirus disease 2019 (COVID-19) have suggested that the disease severity and lung injuries are related to the interaction of tissue-resident memory-like Th17 cells (T_RM_17 cells) with lung macrophages and cytotoxic CD8+T cells. High serum IL-17A and GM-CSF levels in COVID-19 patients are associated with more severe clinical courses (6). Overall, lung T_RM_17 cells are potential coordinators of excessive inflammation in severe COVID-19 (6). Patients recovering from COVID-19 acquire T_RM_ cells with Th1 phenotype against COVID-19 (65). Neutrophils attracted to the site of infection secret IL-17A and stimulate lung epithelial cells to express CCL20. The expression of CCL20 recruits natural Th17 (nTh17) cells to the infected site. In the presence of IL-23, nTh17 are converted to T_RM_ cells (66). COVID-19 prevents the T_RM_ cells from remaining ex-Th17 cells and exerting Th1-like immunity effects (42). Previous studies showed in RSV-immune mice, T_RM_ cells enhanced respiratory syncytial virus clearance, indicating CD8+T_RM_ cells can enhance resistance against secondary RSV infection (43, 44). In RSV-immune mice, CD69 co-expressed heavily with CD38, consistent with its role as an early activation marker. Some CD4+CD69+T cells also expressed integrin CD103, and permanent memory CD4+T cells were enriched in airways. As the infection progressed, these T_RM_ cells were enriched in infection site with increased CXCR3 expression (45). Similarly, T_RM_ cells protect the human body from influenza viruses by producing large amounts of IFN-γ and TNF-α (50). When encountered with influenza A virus, dendritic cells with high expression of IRF4 can effectively promote the production of CD8+T_RM_ cells, thus reducing infection severity (67). PPAR-γ expression accelerates the establishment of CD8+TRM cells, suggesting that PPAR-γ is a positive regulatory factor for T_RM_ cells. Moreover, PPAR-γ deficiency reduces the number of alveolar macrophages residing in tissues during pulmonary infections, indicating that alveolar macrophages might be negative regulators of CD8+T_RM_ cells and could limit the establishment of T_RM_ cells (51).

### Antibacterial Effect

Non-homologous bystander activation can trigger the sensory and alerting functions of lung CD8+T_RM_ cells (68). Unlike memory CD8+T cells in circulating blood, CD8+T_RM_ cells located in the lung parenchyma can rapidly synthesize IFN-γ after the inhalation of heat-killed bacteria or bacterial products, a process-driven by exposure to IL-12/IL-18 (69). Bacterial infection of respiratory tract leads to bystander activation of pulmonary T_RM_ cells, enhancement of the recruitment of neutrophils to the airway and reduction of the severity of bacterial pneumonia (69). These activations suggest that T_RM_ cells innately amplify inflammatory responses and participate in non-homologous responses to bacterial infections (68). Lung CD4+T_RM_ cells remodel epithelial responses to accelerate neutrophil recruitment during pneumonia. During heterotypic immunity, CD4+T cells upregulate CXCL5 and drive neutrophil recruitment in the lung (70). In mice infected with Bordetella pertussis, T_RM_ cells produced IL-17 and IFN-γ, recruiting neutrophils and preventing nasal colonization (46–49). In addition, uninfected mice acquired immunity after receiving adoptive transferred CD4 T cells isolated from either lungs or spleens of convalescent mice (71). Following mucosal znBAZ vaccination, lung CD8+ T_RM_ cells exhibit superior protection against Brucella infections. Mucosal znBAZ immunization induces CD103+ and CD103- CD8+ T_RM_ cells expressing CXCR3^lo^ and CXCR3^hi^ phenotypes in the lung parenchyma and airways, respectively. These CXCR3-expressing CD103+ and CD103-CD8+T_RM_ cells are not depleted by anti-CD8 mAb treatment (52).

### Other Effect

T_RM_ cells also increase resistance to parasite invasions. Both the percentage and absolute numbers of lung CD4+ and CD8+ cells increase after Schistosoma. japonicum infection (72). CD103-expressing pulmonary CD4+ and CD8+ T cells play essential roles in mediating granulomatous inflammation induced by S. japonicum infection (72).

## Lung T_RM_ Cells in Anti-Tumor Immunity

Approximately 85% of lung cancers worldwide are non-small cell lung cancer (NSCLC), of which lung adenocarcinoma (LUAD) and lung squamous cell carcinoma (LUSC) are the most common (73). Growing evidence suggested that in human solid tumors, tumor-associated lymphocytes in NSCLC may comprise the function of T_RM_ cells (25). CD8+T_RM_ cells in the tumor microenvironment (TME) are a homogeneous CD103+CD49+CD69+ population expressing T-bet, porylated (p)STAT-3, and Aiolos transcription factors and a subset of these cells produces IFN-γ and IL-17. In patients with NSCLC, CD8+T_RM_ cells overexpress several T cell inhibitory receptors and exhaustion surface markers and co-express PD-1 and CD39, implying that they are enriched in activated tumor-antigen reactive T cells (74). Cytotoxic CD8+T lymphocytes (CTLs) in NSCLC with a high level of CD103 display enhanced cytotoxicity and proliferation, suggesting a robust anti-tumor immune response in human lung cancer (41). Compared to T cells from adjacent and tumor-free lung tissues, these cells exhibit more significant heterogeneity in the expression of molecules associated with T cell antigen receptor (TCR) activation and immune checkpoints such as 4-1BB, PD-1, TIM-3. However, in human lung cancer, far from being exhausted, PD-1-expressing T_RM_ cells in tumors are clonally expanded and enriched for transcripts linked to cell proliferation and cytotoxicity (12).

The origination, infiltration, and differentiation of T_RM_ in NSCLC is still unclear. O’Brien and colleagues’ model speculated that in patients with early-stage NSCLC, T_EM_ cells encountered antigens during tumor formation and were converted to CD103+T_RM_ cells that exerted anti-tumor activity (75). However, due to a variety of factors associated with tumor growth in TME, the tumors might not be eliminated. These factors, combined with the chronic antigen stimulation, may trigger an exhaustion program characterized by increased Eomes and CD39 expression. The presence of B7-H4 in tumors or other TME stromal cells might upregulate Eomes expression in T cells. As a tumor grows, this exhaustion program may dominate T_RM_ cells, causing increasing TILs hypofunctionality.

In addition, transcription factors may play a role. Patients suffering from advanced-stage NSCLC exhibit a progressive decrease of NFATc1 in tumor cells and TILs decrease progressively (76). Some CD103+T_RM_ cells may lead to decreased function and cytotoxicity of CD8+T cells, a phenomenon observed in the lungs of tumor-bearing NFATc1^ΔCD4^ mice, likely promoted by decreased IL-2 in the absence of NFATc1 (77). Runx3 also plays an important part in the differentiation of T_RM_ in NSCLC (78). The Runx3 is required for optimal T_RM_ cell differentiation in the lung parenchyma and maximal expression of granzyme B in T_RM_ cells. Several tissue-residency signature genes are upregulated in Runx3-overexpressing cells and downregulated in Runx3-deficient cells. Conversely, circulating memory cell signatures are enriched in Runx3-deficient cells and depleted from Runx3-overexpressing cells.

Other immune cells such as M1^hot^ tumor-associated macrophages (TAM) can boost the infiltration and survival of T_RM_ cells in patients with NSCLC (32). M1^hot^ TAMs may recruit T_RM_ cells *via* CXCL9 expression and sustain them by producing more essential fatty acids on which T_RM_ cells depend. Monocytes acquire the ability to prime T_RM_ cells *via* IL-10-mediated TGF-β release. IL-10 plays a negative regulatory role in the immune system per classical theory, but it has been found that CD103 was significantly upregulated, and T cells transform into T_RM_ cells when influenced by IL-10 (33). Significantly upregulated CD103 leads to more T cells transforming into T_RM_ cells. Therefore, IL-10-mediated TGF-β signaling may have a critical role in post-vaccination T_RM_ generation (33).

Moreover, chemokine receptors and cytoskeleton proteins contribute to T_RM_ cell infiltration. The focal adhesion-associated protein paxillin binding to the CD103 cytoplasmic tail triggers αEβ7 integrin outside-in signaling that promotes the migration and functions of CD8+T cells (79). This binding process explains the more favorable prognosis associated with more retention of T_RM_ cells in TME (79). In both mice and human lungs, CXCR6 is expressed on the surface of T_RM_ cells with the action of intrapulmonary antigens, aiding the migration of T_RM_ cells from pulmonary interstitium to TME and maintaining the T_RM_ cell pool (80), whereas memory CD8+T cells of the spleen do not express this receptor (81).

**Figure 2:**
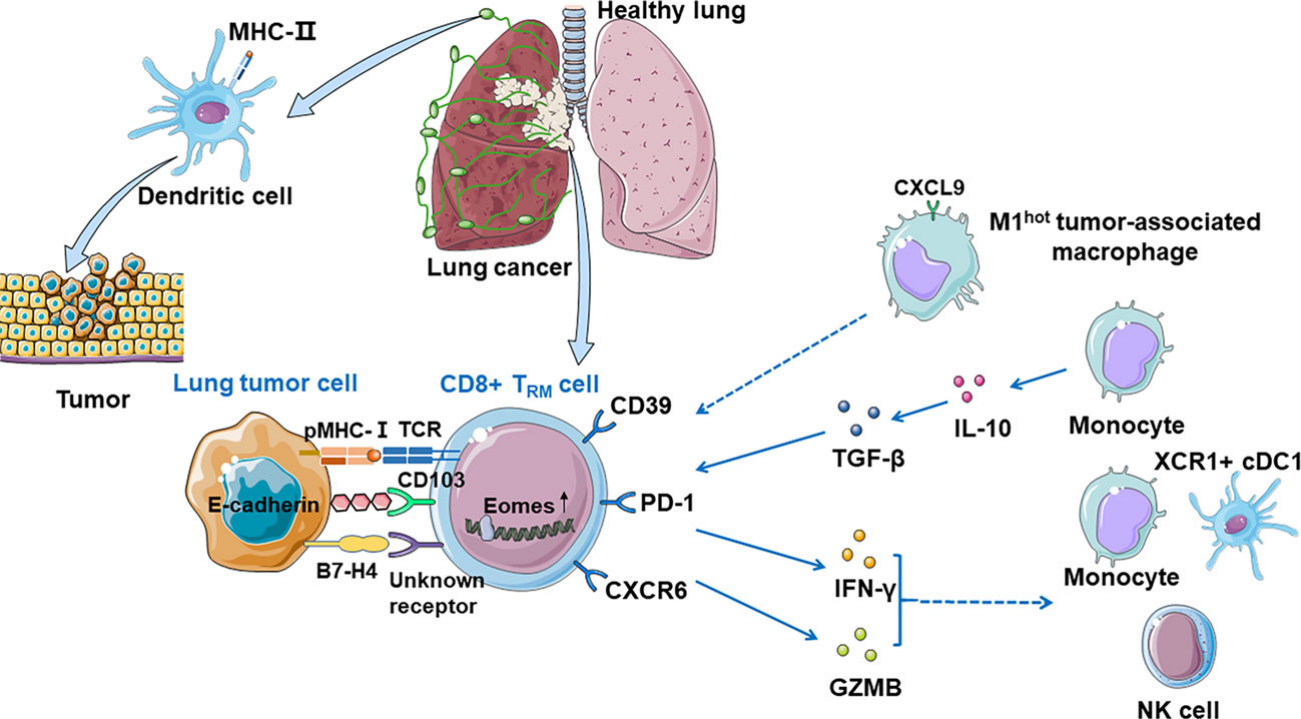
CD8+ T_RM_ cells in anti-tumor immunity. Dendritic cells present tumor antigens in the tumor-draining lymph nodes and then migrate to the tumor where they recognize their cognate antigens and expand, priming CD8+ T cells significantly. Tumor-associated CD8+ T_RM_ cells can be identified by CD39 and CD103, and CD103 promotes immunologic synapse by binding to E-cadherin on tumor cells. T_RM_ cells which express PD-1 are expanded and enriched for transcripts linked to cell proliferation and cytotoxicity. CXCR6 is expressed on the surface of T_RM_ cells when exposed to tumor antigens, transferring T_RM_ cells from pulmonary interstitium to tumor microenvironment, and maintaining the T_RM_ cell pool. M1^hot^ TAMs recruit T_RM_ cells *via* CXCL9 expression and sustain them by making more essential fatty acids on which T_RM_ cells depend. Monocytes prime T_RM_ cells *via* IL-10-mediated TGF-β release which increases the number of local T_RM_ cells. CD8+ CD103+ T_RM_ cells can also produce GZMB and IFN-γ, which recruits monocytes, NK cells, and XCR1+ cDC1 to the tumor site. B7-H4 on tumor cells might upregulate Eomes in T cells, which may cause growing TILs hypofunctionality.

Duhen et al. proposed a model in human solid tumors associating T_RM_ cells with tumor growth: CD8+ T cells are primed by dendritic cells presenting tumor antigens within the tumor-draining lymph nodes and then migrate to the tumor where they recognize the cognate antigens then clonally expand (25). The consequence of this TCR activation in a TGF-β-rich environment is the upregulation of CD39 and CD103 on CD8+ TILs. CD103 expressed on some T_RM_ cells may promote immunologic synapse by binding to E-cadherin on tumor cells (82). Activation of these cells also leads to the downregulation of the proteins that are essential for T-cell recirculation, retaining CD8+ TILs within the tumor. In human lung cancer, there are many important anti-tumor costimulatory molecules such as SIRPG and KIR2DL4 on the surface of T_RM_ cells, which help the CTLs kill tumor cells (41). In human, CD103+ T_RM_ cells can also produce granzyme B (GZMB) and IFN-γ, which can restrict tumor cell growth and metastasis by inducing fibronectin production, make antigens available to prime for the priming of new tumor-specific T cells, and enhance recruitment of monocytes, NK cells, and XCR1+ cDC1 to the tumor site (9). However, repetitive TCR stimulations of the CD8+ TILs impair effector function, mediate immune escape, and ultimately tumor progression. CD103+CD39+CD8+T_RM_ cells efficiently kill autologous tumor cells in an MHC-class I-dependent manner. Additionally, the content of cytokine and receptor function influence immune functions (**Figure 2**).

The infiltration of CD8+ T cells in solid tumors is a favorable prognostic marker (83). In the tumors that exhibited a high level of infiltrated CD8+ T cells, the proliferation of CD103+ T cells was correlated with improved long-term survival, indicating that infiltration of CD8+CD103+ T_RM_ cells is a favorable prognostic marker (84). Another study suggested that high intratumoral but not stromal CD103+ TILs were associated with better overall survival in patients with resected LUSC, another significant prognostic implication of CD103 expression in TILs in human LUSC (85). Moreover, CD103 and E-cadherin interaction play a vital role in granule polarization and exocytosis, enhancing recruitment and retention of tumor-antigen-specific TILs in human NSCLC. In human NSCLC, CD28H is mainly expressed in T_RM_ cells and is thus associated with improved tumor prognosis (86). However, other studies conversely demonstrated opposite results that B7-H5 (the ligand of CD28H) was expressed in more than 60% of cases of NSCLC and was associated with worse prognosis. Hence, the expression patterns of CD103 in TILs of NSCLC and the associated prognostic implications are significant and merit further investigation.

In T_RM_ cells in human NSCLC tissues, there are several dysfunctional subtypes, such as NKG2A+CD8+ T cells (87). NKG2A is an inhibitory receptor of both T cells and natural killer (NK) cells. Persistent activation causes T cells and NK cells to express NKG2A and may lead to chronic infection and cancer. Tumor-infiltrating NKG2A+CD8+T cells form the predominant subset of NKG2A+cells in human lung cancer (87). Blockading NKG2A may promote anti-tumor immunity by unleashing dysfunctional CD8+T cells in tumors, and targeting NKG2A+CD8+T cells is a promising approach for future anti-lung cancer immunotherapy.

While numerous studies have reported that the presence of T_RM_-like CD8+T cells in human NSCLC is a favorable prognosis (77), the role of CD4+TILs with a shared phenotype remains unclear. As CD4+T_RM_ cells exhibit phenotypic and functional heterogeneity, different subsets are expected to play different and even opposite roles in TME. CD4+T_RM_ cells are known to be essential for cytotoxic programming of CD8+T cells, and they can also suppress tumor growth through secretion of IFN-γ or direct killing tumor cells in human NSCLC (88).

## Potential Therapeutic Strategies Based on T_RM_ Cells

Given the remarkable roles of T_RM_ cells in lung diseases, increasing T_RM_ production or reactivating suppressed T_RM_ cells may be a valuable therapeutic strategy (**Table 3**). It is believed that in lung diseases without medical intervention, T_RM_ cells play a less critical role because their function is inhibited and disabled in the focal microenvironment (88). Therefore, current researches focus on reactivating T_RM_ cells that have adapted to the disease microenvironment, increasing the load of T_RM_ cells in the lesions, and mediating immune responses such as cytotoxicity and conditioning, to kill pathogens or slow down disease progression (**Table 4**).

**Table 3:** Strategies to improve the efficacy of vaccines and adoptive cell therapies by targeting T_RM_ cells.

Strategies	Examples	Ways to improve	References
Transcription Factors	Runx3, Bhlhe40, BATF, NAB1, NAB2	Up-regulation	(40, 41, 78)
Cytokines	TGF-β、IL-10	Increment	(33, 59)
Leukocyte surface antigen	CD39, CXCR6, PPAR-γ, SIRPG, KIR2DL4	Activation	(41, 51, 81)
Cells	M1^hot^ TAM cells, Reticular fibroblasts, Dendritic cells with high expression of IRF4	Activation	(32, 55, 67)
	Alveolar macrophage	Inhibition	(51)

**Table 4:** Molecules in mice or/and humans regulating lung T_RM_ cells.

The process lung T_RM_ cells participate in	Species	Regulatory molecules	References
Anti-tumor immunity	Mouse	Runx3, NFATc1, CXCR6, TGF-β	(40, 41, 78)
	Human	Eomes, CD39, CXCL9, paxillin, TGF-β, SIRPG, KIR2DL4	(25, 32, 41, 74, 75, 79)
Positive role in infection	Human	ICOSL, CD40, IL-6, IL-10	(55, 59)
Negative role in infection	Mouse	TGF-β, IL-5, IL-13	(15, 89)
Antivirus immunity	Mouse	CD69, CD38, CD103, CXCR3, IFN-γ, IRF4, PPAR-γ	(45, 50, 51, 67)
	Human	IL-17A, CCL20, IL-23	(42)
Antibacterial Immunity	Mouse	IFN-γ,IL-12,IL-17,IL-18, CXCL5, CXCR3	(46–49, 68, 70)
Association with asthma	Mouse	CD44, ST2, IFN-γ, perforin, granules	(18, 53, 54)

### T_RM_ Cells and Neoadjuvant Chemotherapy

T_RM_ cells may be involved in varied NSCLC therapies. Neoadjuvant chemotherapy was one modality in the treatment of resectable NSCLC. The beneficial effects of neoadjuvant chemotherapy might be mediated partially by CD8+CD103+ mediated tumor cell killing (13). With neoadjuvant chemotherapy, more infiltration of CD4+CD103+PD-1 T_RM_ cells at the time of surgery was associated with longer overall survival. Moreover, T_RM_ cells could be of great importance in TME and in cancer immune checkpoint blockade immunotherapy. In both mice model and human, dual anti-PD-L1/anti-4-1BB immunotherapy increased the number of intratumoral CD103+CD8+ T cells and altered their distribution (90). Administration of PD-L1 mAb and 4-1BB mAb further increased the cytolytic capacity of CD103+CD8+T cells. Collectively, infiltrated CD103+CD8+T cells served as a potential effector T cell population. Combining 4-1BB agonism with PD-L1 blockade may increase tumor-infiltrated CD103+CD8+T cells, facilitating tumor regression. It is also reported that CD103+CD8+T_RM_ cells could be considered potential biomarkers when selecting patients that may benefit from immune checkpoint blockade immunotherapy in patients with multiple primary lung adenocarcinoma after neoadjuvant immunotherapy (91). Yet more evidence is required to determine the clinical practice of potential the therapeutic strategies based on T_RM_ cells, as a more favorable indicator of prognosis or a target of immune therapy.

Several treatments may potentially activate or increase the number of T_RM_ cells. One possible treatment involves promoting the separation and trans-differentiation of T cells to T_RM_ cells in TME, which could inhibit tumor progression. At the same time, in murine models, apoptosis induced by IR increases the number of newly infiltrated T cells and converts them into T_RM_ cells, producing an inflammation-like effect that may assist immunotherapy (92).

### T_RM_ Cells and Radiotherapy

Since T_RM_ cells have a unique survival advantage in radiotherapy, a RT-PD1-MerTK triple therapy based on radiotherapy may also be effective. Promoting T_RM_ cell production from other sources such as traditional radiotherapy may be an equally valuable potential treatment. T_RM_ cells have stronger radiation resistance than tumor cells, and efficient infrared irradiation (IR) makes pre-existing T_RM_ cells survive and mediates the anti-tumor effect of T_RM_ cells (93). In murine models, compared with traditional radiotherapy, anti-PD-1 therapy relieves the inhibitory effect on immune cells such as T_RM_ cells, while anti-MerTK can transform apoptosis caused by radiation into cell necrosis and turn macrophages near tumors from M2 to M1 and reduce tumor load (94). This triple therapy could increase the content of CTLs and promote the differentiation of T_RM_ cells, improving the anti-tumor effect. Adding anti-PD1 and anti-MerTK to radiation could significantly upregulate CD8+CD103+TRM at the abscopal tumors, suppress the abscopal tumor growth and extended the survival rate (95). As for epigenetic and metabolic regulation, a treatment scheme for TA/AC may be considered. TA, or microtubule inhibitor A, is a histone deacetylase inhibitor that can promote the production of IFN- γ in Bhlhe40+CD8+T_RM_ cells (96). Acetic acid (AC) can be used as the substrate of acetyl-CoA synthesis, which is independent from the tricarboxylic acid (TCA) cycle and promotes histone acetylation and cytokine production in Bhlhe40+CD8+ T_RM_ cells (97). The combination of TA and AC can promote tissue retention and functional differentiation of T_RM_ cells (40). TA/Ac treatment not only enhances Bhlhe40−/− CD8+ T cell effector and resident gene expression but also promotes the expression of these genes in WT CD8+ T cells, indicating appropriate combinations of epigenetic modifiers with certain metabolites may represent promising approaches for maximally reinvigorating tissue or tumor-resident CD8+ T cell antiviral or antitumor activities.

### T_RM_ Cells and Vaccines

Another treatment approach involves the induction of persistent T_RM_ cells by vaccines. Phosphatidylserine liposomes are excellent antigen carriers, which can be combined with polyconic adjuvants for the development of new BCG vaccines (71). Because anti-cytomegalovirus response is one of the most powerful and persistent cellular immune responses observed in human bodies, cytomegalovirus is a possible effective T_RM_-cell-inducing vaccine vector (98). Murine models show that other peptide nanofibers with strong immunogenicity may likewise improve the immune response (99), particularly with combined polypeptide antigen and adjuvant (33). Continuous stimulation with local homologous antigens can increase T_RM_ cell population, and zymosan used as an adjuvant could transform CD8+T cells into T_RM_ cells in the absence of antigens. Mice models indicate that adding zymosan adjuvant to a possible vaccine may moderate local inflammation as well as greatly enhance the production of T_RM_ cells (100). Similarly, combining ovalbumin antigen and CpG DNA adjuvant hybridized pH-responsive substances can increase the T_RM_ cells response range and lifespan. This combination can also activate antigen-presenting cells (APCs), and stimulate continuous T_RM_ cell production in mice (101).

Intranasal vaccine administration induces T_RM_ cells in the lung (11). Triggering an appropriate inflammatory response in the immune process may allow T_RM_ cells to bypass antigen recognition. Lung T_RM_ cells are most effectively induced at the memory stage of basic vaccines in murine models (99). In-depth analysis of the phenotypes of the locally induced CD8+T cells showed that after vaccination, T_RM_ cells and CD8+T cells coexist as effector phenotypes and that T_RM_ cells play an important role. Indeed, at the peak of the local immune response, concentrations of T_RM_ are 10-fold higher than those of effector CD8+T cells, and only the T_RM_ cells population persist locally after 30 days. Even when effector CD8+T cells are no longer detectable, tumor resistance is still observed (11).

The CXCR6-CXCL16 axis demonstrably governs the growth of NSCLC in the migration of CD8+ resident memory T cells in lung mucosa after vaccination. CXCR6 deficiency impairs cancer vaccine efficacy and CD8+ resident memory T-cell recruitment in lung tumors (80). Interestingly, intranasal vaccination induces higher and more sustained concentrations of CXCL16 than intramuscular vaccination, particularly compared with other chemokines in the bronchoalveolar lavage fluid and pulmonary parenchyma in both mice and human (81).

Despite it is observed that vaccines can promote the T_RM_ cells population, retention and function and then enhance the anti-tumor immunity both in mice and human, the efficiency and safety of tumor-related vaccines remains unclear thus require further investigations.

## Lung T_RM_ and Immunopathology

In certain conditions, lung T_RM_ cells may cause excessive inflammatory responses and tissue fibrosis (**Table 4**). After acute influenza infection, abnormal reactivation of T_RM_ cells in the lung may likewise cause lung tissue changes and fibrosis (102). In elderly mice, CD8+ T_RM_ cells can be abnormally deposited in the lung due to overexpression of TGF-β-related genes (103). Low responsive T_RM_ cells not only fail to perform an immune function but may also lead to chronic inflammation and the sequelae of fibrosis (89). CD69^hi^CD103^lo^CD4+ T_RM_ cells produce effector cytokines and promoted the inflammation and fibrotic responses induced by chronic exposure to Aspergillus fumigatus (15). Studies have shown that pathogenic immune cells like CD69^hi^CD103^lo^CD4+T_RM_ cells enhance the secretion of IL-5 and IL-13, which can cause excessive pulmonary inflammation and fibrosis. By contrast, CD69^hi^CD103^hi^CD4+TRM cells can improve the fibrosis reaction caused by pulmonary inflammation and reduce lung injury, indicating that lung CD4^+^ T_RM_ cells play crucial roles in the pathology of chronic lung inflammation, and CD103 expression defines pathogenic effector and immunosuppressive T_RM_ cell subpopulations in the the lung (15).

### Association With Asthma

Th2-T_RM_ cells are associated with asthma. These cells release cytokines that recruit eosinophils and sustain mast cells in the airway, leading to an inflammatory response. Th2-T_RM_ cells expressed with high levels of CD44 and ST2 have been observed in lung tissues and can retain allergen memory throughout the life of a host organism (53). Re-exposure to a known allergen causes Th2-T_RM_ cells to proliferate near the airway, producing type 2 cytokines that enhance eosinophil activation and promote peribronchial inflammation (104). Together with circulating memory Th2 cells, they perform non-redundant functions in asthma induction (18, 53, 54). Although these T_RM_ cells eliminate invasive pathogens, the release of pro-inflammatory factors (such as IFN-γ, perforin, and granulose) may damage normal cells, leading to lung damage, emphysema, or fibrosis.

#### Participation in Acute Cellular Rejection (ACR) After Lung Transplantation

T cells are mediators of acute cellular rejection (ACR) after lung transplantation (105). The role of pulmonary T_RM_ cells in ACR in lung transplantation remains uncertain. Longitudinal analysis of lung transplant recipients has indicated that T_RM_ cells from recipients gradually formed T_RM_ phenotypes approximating healthy people after 6 months allograft, while donor T cells persisted in the form of T_RM_. The increase in the proportion of recipient-derived T_RM_ cells was associated with ACR, suggesting that T_RM_ cells may influence the inflammatory environment of lung allograft after transplantation (2).

## Conclusion

Human lung T_RM_ cells, whether CD8+ or CD4+, persist in lung tissues for decades of human life. The essential role of lung T_RM_ cells is maintaining tissue homeostasis when facing viruses, antigens, and pathogens encountered through respiration, and may also be important in tumor surveillance. Lung T_RM_ cells can also promote pathologic inflammation, inducing chronic airway inflammatory changes leading to asthma and fibrosis. Similarly, lung T_RM_ cells from infiltrating recipient T cells in transplantation may mediate allograft immunopathology and promote lung damage. More comprehensive understanding of the induction and maintenance of T_RM_ cells by cancer vaccines or other immunotherapeutic approaches may provide insights into the innovation of immunotherapies of lung diseases.

## Author Contributions

CC provided the concepts and ideas of the article. RY, JY, ZJ, and JL performed literature search and wrote the manuscript’s first draft. CC, FW, XH, and RY performed a critical revision of the first draft and the final editing of the manuscript. All authors contributed to the article and approved the submitted version.

## Funding

This study was funded by the China National Natural Science Foundation No. 81902351, the Hunan Provincial Natural Science Foundation (No. 2020SK53419, 2021JJ30926 and No. 2019JJ50953), Hunan Provincial Key Area R&D Program NO. 2019SK2253, CSCO Cancer Research Foundation (CSCO-Y-young2019-034 and CSCO-2019Roche-073), and the Changsha Municipal Natural Science Foundation NO. kq2014246.

## Conflict of Interest

The authors declare that the research was conducted in the absence of any commercial or financial relationships that could be construed as a potential conflict of interest.

## Publisher’s Note

All claims expressed in this article are solely those of the authors and do not necessarily represent those of their affiliated organizations, or those of the publisher, the editors and the reviewers. Any product that may be evaluated in this article, or claim that may be made by its manufacturer, is not guaranteed or endorsed by the publisher.
